# Dichlorido[1-(2,6-dimethyl­phenyl­imino)-1,2-diphenyl­propan-2-ol-κ^2^ *N*,*O*]palladium(II) methanol monosolvate

**DOI:** 10.1107/S1600536811037986

**Published:** 2011-09-30

**Authors:** Feng-Shou Liu, Ying-Tang Huang, Dong-Sheng Shen, Hua-Gang Yao

**Affiliations:** a School of Chemistry and Chemical Engineering, Guangdong Pharmaceutical University, Zhongshan, Guangdong 528458, People’s Republic of China

## Abstract

The title compound, [PdCl_2_(C_23_H_23_NO)]·CH_3_OH, was obtained by the reaction of 1-(2,6-dimethyl­phenyl­imino)-1,2-diphenyl­propan-2-ol and palladium chloride in methanol. The Pd atom is four-coordinated by the O atom of a tertiary alcohol, the imine N atom of the hy­droxy­limine part of the bidentate ligand and by two chloride ions, forming a nearly square-planar geometry. The complex mol­ecule and the uncoordinated methanol mol­ecule are connected *via* an O—H⋯O hydrogen bond.

## Related literature

For transition metal complexes of (*N*,*O*)-bidentate ligands, see: Skrolkhod *et al.* (2002 ▶); Macchioni *et al.* (2002 ▶); Binotti *et al.* (2004 ▶); Zuccaccia *et al.* (2006 ▶). Complexes with group IV metals with (*N*,*O*)-bidentate ligands, which form six-membered rings, have been widely used in the production of polyethyl­ene with high mol­ecular weight and relative narrow mol­ecular weight distribution, see: Jia & Jin (2009 ▶); Mu *et al.* (2009 ▶). For the use of palladium complexes in Suzuki–Miyaura cross-coupling reactions, see: Lai *et al.* (2005 ▶).
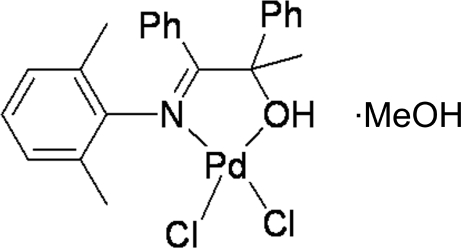


## Experimental

### 

#### Crystal data

[PdCl_2_(C_23_H_23_NO)]·CH_4_O
*M* *_r_* = 538.79
Monoclinic, 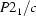

*a* = 10.943 (3) Å
*b* = 19.770 (6) Å
*c* = 14.230 (3) Å
β = 129.232 (13)°
*V* = 2384.6 (11) Å^3^
*Z* = 4
Mo *K*α radiation
μ = 1.02 mm^−1^
*T* = 296 K
0.25 × 0.20 × 0.10 mm

#### Data collection

Bruker SMART APEXII CCD diffractometer
Absorption correction: multi-scan (*SADABS*; Sheldrick, 2001 ▶) *T*_min_ = 0.779, *T*_max_ = 0.901
35358 measured reflections
4166 independent reflections
3418 reflections with *I* > 2σ(*I*)
*R*_int_ = 0.036

#### Refinement

*R*[*F* ^2^ > 2σ(*F* ^2^)] = 0.042
*wR*(*F* ^2^) = 0.082
*S* = 1.01
4166 reflections
280 parameters
H atoms treated by a mixture of independent and constrained refinement
Δρ_max_ = 0.50 e Å^−3^
Δρ_min_ = −0.42 e Å^−3^

### 

Data collection: *APEX2* (Bruker, 2001 ▶); cell refinement: *SAINT-Plus* (Bruker, 2001 ▶); data reduction: *SAINT-Plus*; program(s) used to solve structure: *SHELXS97* (Sheldrick, 2008 ▶); program(s) used to refine structure: *SHELXL97* (Sheldrick, 2008 ▶); molecular graphics: *SHELXTL* (Sheldrick, 2008 ▶); software used to prepare material for publication: *SHELXTL*.

## Supplementary Material

Crystal structure: contains datablock(s) global, I. DOI: 10.1107/S1600536811037986/kp2349sup1.cif

Structure factors: contains datablock(s) I. DOI: 10.1107/S1600536811037986/kp2349Isup2.hkl

Additional supplementary materials:  crystallographic information; 3D view; checkCIF report

## Figures and Tables

**Table 1:** Selected bond lengths (Å)

Pd1—O1	2.019 (3)
Pd1—N1	2.032 (3)
Pd1—Cl1	2.2588 (13)
Pd1—Cl2	2.2859 (13)

**Table 2:** Hydrogen-bond geometry (Å, °)

*D*—H⋯*A*	*D*—H	H⋯*A*	*D*⋯*A*	*D*—H⋯*A*
O1—H7⋯O2^i^	0.76 (6)	1.80 (6)	2.535 (5)	164 (7)

Symmetry code: (i) 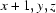.

**Fig. 1.**
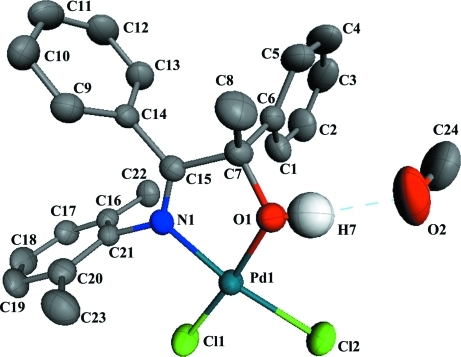
The molecular structure of the title complex showing displacement ellipsoids drawn atthe 30% probability level. Hydrogen bond is drawn as dashed line. H atoms not related to the hydrogen bonding are omitted. Symmetry code:(i) x + 1, y, z

## supplementary crystallographic information

### Comment

Recently, the bidentate (N, O) ligand such as salicylaldimine and hydroxylimine have drawn much attention owing to their valuable applications in the fields of catalysis. These bidentate ligands can be modified by tuning the substituents. Therefore, different steric and electronic properties are achieved easily. Various transition metal complexes (Skrolkhod *et al.* 2002; Macchioni *et al.* 2002; Binotti *et al.* 2004; Zuccaccia *et al.* 2006) have been developed. Especially, complexes with metals of the  group IV containing (N, O) ligands have been widely used to produce polyethylene with high molecular weight and relative narrow molecular weight distribution (Mu *et al.* 2009; Jia *et al.* 2009). Moreover, the palladium complexes also have been applied for Suzuki-Miyaura cross-coupling reaction (Lai *et al.* 2005). We report herein on the synthesis and structure of the title compound. The palladium atom is four-coordinated by the oxgen atom o a tertiary alcohol and imine nitrogen atom of the bidentate hydroxylimine ligand, and by the two chloride ions, forming a nearly square-planar geometry (Fig. 1, Table 1). The solid-state structure showes a noncentrosymmetric palladium complex with one uncoordinated methanol solvated molecule. The complex molecule and the uncoordinated methanol molecule are connected *via* O—H···O hydrogen bond (Table 2).

### Experimental

A 100 ml round-bottle was charged with palladium chloride (0.177 g, 1 mmol), 1-(2,6-dimethylphenylimino)-1,2-diphenylpropan-2-ol (0.329 g, 1 mmol), and methanol (20 mL). After the mixture was stirred for 24 h at room temperature, the methanol was removed under reduced pressure. The red crystals suitable for X-ray diffraction wwere prepared by slow evaporation of a solution of the title compound in methanol at room temperature.

### Refinement

All H atoms were positioned geometrically with C—H = 0.93Å and allowed to ride during subsequent refinement with *U*_iso_(H)=1.2*U*_eq_(C)

### Crystal data

**Table:**

[PdCl_2_(C_23_H_23_NO)]·CH_4_O	*F*(000) = 1096
*M**_r_* = 538.79	*D*_x_ = 1.501 Mg m^−^^3^
Monoclinic, *P*2_1_/*c*	Mo *K*α radiation, λ = 0.71073 Å
Hall symbol: -P 2ybc	Cell parameters from 4166 reflections
*a* = 10.943 (3) Å	θ = 2.3–25.5°
*b* = 19.770 (6) Å	µ = 1.02 mm^−^^1^
*c* = 14.230 (3) Å	*T* = 296 K
β = 129.232 (13)°	Block, yellow
*V* = 2384.6 (11) Å^3^	0.25 × 0.20 × 0.10 mm
*Z* = 4	

### Data collection

**Table:**

Bruker SMART APEXII CCD diffractometer	4166 independent reflections
Radiation source: fine-focus sealed tube	3418 reflections with *I* > 2σ(*I*)
graphite	*R*_int_ = 0.036
φ and ω scans	θ_max_ = 25.0°, θ_min_ = 2.1°
Absorption correction: multi-scan (*SADABS*; Sheldrick, 2001)	*h* = −13→11
*T*_min_ = 0.779, *T*_max_ = 0.901	*k* = −23→18
35358 measured reflections	*l* = −16→16

### Refinement

**Table:**

Refinement on *F*^2^	Primary atom site location: structure-invariant direct methods
Least-squares matrix: full	Secondary atom site location: difference Fourier map
*R*[*F*^2^ > 2σ(*F*^2^)] = 0.042	Hydrogen site location: inferred from neighbouring sites
*wR*(*F*^2^) = 0.082	H atoms treated by a mixture of independent and constrained refinement
*S* = 1.01	*w* = 1/[σ^2^(*F*_o_^2^) + (0.*P*)^2^ + 10.0286*P*] where *P* = (*F*_o_^2^ + 2*F*_c_^2^)/3
4166 reflections	(Δ/σ)_max_ = 0.001
280 parameters	Δρ_max_ = 0.50 e Å^−^^3^
0 restraints	Δρ_min_ = −0.42 e Å^−^^3^

### Special details

**Table:**

Geometry. All e.s.d.'s (except the e.s.d. in the dihedral angle between two l.s. planes) are estimated using the full covariance matrix. The cell e.s.d.'s are taken into account individually in the estimation of e.s.d.'s in distances, angles and torsion angles; correlations between e.s.d.'s in cell parameters are only used when they are defined by crystal symmetry. An approximate (isotropic) treatment of cell e.s.d.'s is used for estimating e.s.d.'s involving l.s. planes.
Refinement. Refinement of *F*^2^ against ALL reflections. The weighted *R*-factor *wR* and goodness of fit *S* are based on *F*^2^, conventional *R*-factors *R* are based on *F*, with *F* set to zero for negative *F*^2^. The threshold expression of *F*^2^ > σ(*F*^2^) is used only for calculating *R*-factors(gt) *etc*. and is not relevant to the choice of reflections for refinement. *R*-factors based on *F*^2^ are statistically about twice as large as those based on *F*, and *R*- factors based on ALL data will be even larger.

### Fractional atomic coordinates and isotropic or equivalent isotropic displacement parameters (Å^2^)

**Table:**

	*x*	*y*	*z*	*U*_iso_*/*U*_eq_	
Pd1	0.81208 (4)	0.748377 (17)	0.18909 (3)	0.03477 (10)	
C1	0.9955 (7)	0.5030 (3)	0.2701 (5)	0.0589 (14)	
H1	1.0476	0.4900	0.3501	0.071*	
C2	0.9958 (8)	0.4603 (3)	0.1929 (7)	0.0767 (19)	
H2	1.0495	0.4193	0.2218	0.092*	
C3	0.9173 (8)	0.4781 (3)	0.0743 (7)	0.0777 (19)	
H3	0.9160	0.4489	0.0224	0.093*	
C4	0.8410 (7)	0.5390 (3)	0.0322 (6)	0.0703 (16)	
H4	0.7896	0.5515	−0.0478	0.084*	
C5	0.8402 (6)	0.5813 (3)	0.1079 (5)	0.0567 (13)	
H5	0.7862	0.6222	0.0778	0.068*	
C6	0.9187 (5)	0.5646 (2)	0.2297 (4)	0.0424 (11)	
C7	0.9203 (5)	0.6152 (2)	0.3113 (4)	0.0402 (10)	
C8	1.0248 (5)	0.5949 (3)	0.4447 (4)	0.0539 (13)	
H8A	1.1329	0.5932	0.4771	0.081*	
H8B	0.9934	0.5512	0.4520	0.081*	
H8C	1.0140	0.6275	0.4890	0.081*	
C9	0.6657 (6)	0.5821 (2)	0.3767 (4)	0.0463 (11)	
H9	0.6972	0.6224	0.4199	0.056*	
C10	0.6062 (6)	0.5303 (3)	0.4025 (5)	0.0566 (13)	
H10	0.5981	0.5360	0.4632	0.068*	
C11	0.5590 (6)	0.4705 (3)	0.3393 (5)	0.0589 (14)	
H11	0.5186	0.4359	0.3567	0.071*	
C12	0.5720 (6)	0.4623 (3)	0.2502 (5)	0.0584 (14)	
H12	0.5397	0.4219	0.2069	0.070*	
C13	0.6327 (6)	0.5134 (2)	0.2244 (5)	0.0496 (12)	
H13	0.6428	0.5071	0.1648	0.060*	
C14	0.6786 (5)	0.5741 (2)	0.2870 (4)	0.0350 (10)	
C15	0.7533 (5)	0.6285 (2)	0.2647 (4)	0.0347 (9)	
C16	0.3992 (5)	0.6785 (2)	0.0569 (4)	0.0368 (10)	
C17	0.2518 (5)	0.6979 (2)	0.0172 (4)	0.0478 (12)	
H17	0.1621	0.6829	−0.0577	0.057*	
C18	0.2358 (6)	0.7390 (3)	0.0864 (5)	0.0562 (13)	
H18	0.1356	0.7506	0.0589	0.067*	
C19	0.3665 (6)	0.7630 (2)	0.1960 (5)	0.0530 (13)	
H19	0.3540	0.7911	0.2419	0.064*	
C20	0.5179 (5)	0.7460 (2)	0.2396 (4)	0.0417 (10)	
C21	0.5297 (5)	0.7016 (2)	0.1693 (4)	0.0320 (9)	
C22	0.4129 (6)	0.6358 (3)	−0.0233 (4)	0.0538 (13)	
H22A	0.4888	0.6555	−0.0282	0.081*	
H22B	0.3122	0.6334	−0.1030	0.081*	
H22C	0.4463	0.5910	0.0100	0.081*	
C23	0.6612 (6)	0.7760 (3)	0.3556 (4)	0.0566 (14)	
H23A	0.7335	0.7405	0.4070	0.085*	
H23B	0.6300	0.7997	0.3963	0.085*	
H23C	0.7115	0.8069	0.3375	0.085*	
C24	0.2604 (8)	0.6212 (4)	0.2878 (7)	0.100 (2)	
H24A	0.3466	0.6387	0.2935	0.150*	
H24B	0.1649	0.6240	0.2053	0.150*	
H24C	0.2809	0.5748	0.3136	0.150*	
Cl1	0.61727 (15)	0.82479 (7)	0.07282 (12)	0.0586 (3)	
Cl2	0.97468 (14)	0.80417 (7)	0.16612 (11)	0.0527 (3)	
N1	0.6871 (4)	0.68523 (17)	0.2129 (3)	0.0337 (8)	
O1	0.9775 (4)	0.67959 (17)	0.3055 (3)	0.0463 (8)	
O2	0.2444 (5)	0.6582 (3)	0.3605 (5)	0.1014 (17)	
H2A	0.3289	0.6759	0.4148	0.152*	
H7	1.049 (8)	0.676 (3)	0.310 (6)	0.09 (3)*	

### Atomic displacement parameters (Å^2^)

**Table:**

	*U*^11^	*U*^22^	*U*^33^	*U*^12^	*U*^13^	*U*^23^
Pd1	0.03196 (17)	0.03511 (18)	0.03475 (17)	0.00029 (15)	0.01993 (14)	0.00178 (15)
C1	0.071 (4)	0.041 (3)	0.079 (4)	0.006 (3)	0.054 (3)	0.009 (3)
C2	0.103 (5)	0.036 (3)	0.124 (6)	0.008 (3)	0.088 (5)	0.002 (3)
C3	0.098 (5)	0.062 (4)	0.108 (6)	−0.020 (4)	0.082 (5)	−0.030 (4)
C4	0.072 (4)	0.085 (5)	0.064 (4)	0.000 (3)	0.048 (3)	−0.014 (3)
C5	0.050 (3)	0.061 (3)	0.062 (3)	0.010 (3)	0.036 (3)	0.001 (3)
C6	0.033 (2)	0.038 (2)	0.055 (3)	0.0008 (19)	0.026 (2)	0.003 (2)
C7	0.034 (2)	0.035 (2)	0.046 (3)	0.0024 (19)	0.023 (2)	0.003 (2)
C8	0.040 (3)	0.060 (3)	0.042 (3)	0.011 (2)	0.016 (2)	0.010 (2)
C9	0.054 (3)	0.039 (3)	0.051 (3)	0.002 (2)	0.035 (3)	0.001 (2)
C10	0.067 (3)	0.058 (3)	0.065 (3)	0.006 (3)	0.051 (3)	0.011 (3)
C11	0.058 (3)	0.045 (3)	0.077 (4)	0.000 (3)	0.044 (3)	0.013 (3)
C12	0.058 (3)	0.040 (3)	0.074 (4)	−0.011 (2)	0.041 (3)	−0.009 (3)
C13	0.051 (3)	0.043 (3)	0.054 (3)	−0.004 (2)	0.034 (3)	−0.005 (2)
C14	0.031 (2)	0.032 (2)	0.040 (2)	0.0052 (18)	0.022 (2)	0.0047 (18)
C15	0.036 (2)	0.036 (2)	0.030 (2)	0.0024 (19)	0.0199 (19)	−0.0007 (18)
C16	0.035 (2)	0.040 (2)	0.033 (2)	0.0008 (19)	0.020 (2)	0.0044 (19)
C17	0.035 (2)	0.053 (3)	0.046 (3)	0.001 (2)	0.021 (2)	0.000 (2)
C18	0.039 (3)	0.059 (3)	0.069 (3)	0.013 (2)	0.034 (3)	0.009 (3)
C19	0.060 (3)	0.050 (3)	0.067 (3)	0.012 (2)	0.049 (3)	0.001 (3)
C20	0.048 (3)	0.039 (2)	0.039 (2)	0.002 (2)	0.028 (2)	0.003 (2)
C21	0.034 (2)	0.031 (2)	0.034 (2)	0.0033 (17)	0.023 (2)	0.0036 (18)
C22	0.050 (3)	0.067 (3)	0.042 (3)	−0.004 (3)	0.028 (3)	−0.009 (2)
C23	0.073 (4)	0.050 (3)	0.050 (3)	0.003 (3)	0.040 (3)	−0.009 (2)
C24	0.071 (5)	0.107 (6)	0.135 (7)	−0.018 (4)	0.071 (5)	−0.005 (5)
Cl1	0.0481 (7)	0.0585 (8)	0.0675 (8)	0.0156 (6)	0.0358 (7)	0.0293 (7)
Cl2	0.0471 (7)	0.0593 (8)	0.0554 (7)	−0.0096 (6)	0.0341 (6)	0.0001 (6)
N1	0.0306 (18)	0.037 (2)	0.0303 (18)	0.0008 (15)	0.0176 (16)	−0.0013 (15)
O1	0.0330 (18)	0.0400 (19)	0.057 (2)	0.0015 (14)	0.0243 (17)	0.0060 (15)
O2	0.041 (2)	0.118 (4)	0.123 (4)	−0.004 (2)	0.041 (3)	−0.033 (3)

### Geometric parameters (Å, °)

**Table:**

Pd1—O1	2.019 (3)	C12—H12	0.9300
Pd1—N1	2.032 (3)	C13—C14	1.386 (6)
Pd1—Cl1	2.2588 (13)	C13—H13	0.9300
Pd1—Cl2	2.2859 (13)	C14—C15	1.500 (6)
C1—C6	1.382 (6)	C15—N1	1.285 (5)
C1—C2	1.388 (8)	C16—C17	1.385 (6)
C1—H1	0.9300	C16—C21	1.386 (6)
C2—C3	1.370 (9)	C16—C22	1.501 (6)
C2—H2	0.9300	C17—C18	1.370 (7)
C3—C4	1.368 (8)	C17—H17	0.9300
C3—H3	0.9300	C18—C19	1.373 (7)
C4—C5	1.369 (7)	C18—H18	0.9300
C4—H4	0.9300	C19—C20	1.395 (6)
C5—C6	1.402 (7)	C19—H19	0.9300
C5—H5	0.9300	C20—C21	1.395 (6)
C6—C7	1.524 (6)	C20—C23	1.504 (6)
C7—O1	1.444 (5)	C21—N1	1.451 (5)
C7—C15	1.521 (6)	C22—H22A	0.9600
C7—C8	1.525 (6)	C22—H22B	0.9600
C8—H8A	0.9600	C22—H22C	0.9600
C8—H8B	0.9600	C23—H23A	0.9600
C8—H8C	0.9600	C23—H23B	0.9600
C9—C14	1.380 (6)	C23—H23C	0.9600
C9—C10	1.382 (6)	C24—O2	1.366 (8)
C9—H9	0.9300	C24—H24A	0.9600
C10—C11	1.372 (7)	C24—H24B	0.9600
C10—H10	0.9300	C24—H24C	0.9600
C11—C12	1.371 (7)	O1—H7	0.76 (6)
C11—H11	0.9300	O2—H2A	0.8200
C12—C13	1.381 (7)		
			
O1—Pd1—N1	78.37 (14)	C14—C13—H13	119.9
O1—Pd1—Cl1	172.93 (11)	C9—C14—C13	119.0 (4)
N1—Pd1—Cl1	96.32 (10)	C9—C14—C15	120.1 (4)
O1—Pd1—Cl2	93.85 (11)	C13—C14—C15	120.7 (4)
N1—Pd1—Cl2	170.77 (10)	N1—C15—C14	124.2 (4)
Cl1—Pd1—Cl2	91.83 (5)	N1—C15—C7	119.1 (4)
C6—C1—C2	120.8 (5)	C14—C15—C7	116.7 (4)
C6—C1—H1	119.6	C17—C16—C21	117.6 (4)
C2—C1—H1	119.6	C17—C16—C22	119.8 (4)
C3—C2—C1	120.4 (6)	C21—C16—C22	122.6 (4)
C3—C2—H2	119.8	C18—C17—C16	121.2 (4)
C1—C2—H2	119.8	C18—C17—H17	119.4
C4—C3—C2	119.9 (6)	C16—C17—H17	119.4
C4—C3—H3	120.0	C17—C18—C19	120.5 (4)
C2—C3—H3	120.0	C17—C18—H18	119.8
C3—C4—C5	120.0 (6)	C19—C18—H18	119.8
C3—C4—H4	120.0	C18—C19—C20	120.8 (4)
C5—C4—H4	120.0	C18—C19—H19	119.6
C4—C5—C6	121.7 (5)	C20—C19—H19	119.6
C4—C5—H5	119.2	C19—C20—C21	117.1 (4)
C6—C5—H5	119.2	C19—C20—C23	120.8 (4)
C1—C6—C5	117.2 (5)	C21—C20—C23	122.0 (4)
C1—C6—C7	123.4 (4)	C16—C21—C20	122.7 (4)
C5—C6—C7	119.4 (4)	C16—C21—N1	119.8 (4)
O1—C7—C15	105.6 (3)	C20—C21—N1	117.2 (4)
O1—C7—C6	109.3 (4)	C16—C22—H22A	109.5
C15—C7—C6	110.4 (4)	C16—C22—H22B	109.5
O1—C7—C8	107.0 (4)	H22A—C22—H22B	109.5
C15—C7—C8	109.7 (4)	C16—C22—H22C	109.5
C6—C7—C8	114.4 (4)	H22A—C22—H22C	109.5
C7—C8—H8A	109.5	H22B—C22—H22C	109.5
C7—C8—H8B	109.5	C20—C23—H23A	109.5
H8A—C8—H8B	109.5	C20—C23—H23B	109.5
C7—C8—H8C	109.5	H23A—C23—H23B	109.5
H8A—C8—H8C	109.5	C20—C23—H23C	109.5
H8B—C8—H8C	109.5	H23A—C23—H23C	109.5
C14—C9—C10	120.2 (5)	H23B—C23—H23C	109.5
C14—C9—H9	119.9	O2—C24—H24A	109.5
C10—C9—H9	119.9	O2—C24—H24B	109.5
C11—C10—C9	120.6 (5)	H24A—C24—H24B	109.5
C11—C10—H10	119.7	O2—C24—H24C	109.5
C9—C10—H10	119.7	H24A—C24—H24C	109.5
C12—C11—C10	119.5 (5)	H24B—C24—H24C	109.5
C12—C11—H11	120.3	C15—N1—C21	121.8 (4)
C10—C11—H11	120.3	C15—N1—Pd1	115.9 (3)
C11—C12—C13	120.5 (5)	C21—N1—Pd1	122.3 (3)
C11—C12—H12	119.8	C7—O1—Pd1	116.4 (3)
C13—C12—H12	119.8	C7—O1—H7	112 (5)
C12—C13—C14	120.2 (5)	Pd1—O1—H7	119 (5)
C12—C13—H13	119.9	C24—O2—H2A	109.5

### Hydrogen-bond geometry (Å, °)

**Table:**

*D*—H···*A*	*D*—H	H···*A*	*D*···*A*	*D*—H···*A*
O1—H7···O2^i^	0.76 (6)	1.80 (6)	2.535 (5)	164 (7)

Symmetry codes: (i) *x*+1, *y*, *z*.
